# Understanding the role of NRF2-regulated miRNAs in human malignancies

**DOI:** 10.18632/oncotarget.1181

**Published:** 2013-08-08

**Authors:** Niraj M Shah, Stuart A Rushworth, Megan Y Murray, Kristian M Bowles, David J MacEwan

**Affiliations:** ^1^ Norwich Medical School, University of East Anglia, Norwich Research Park, Norwich, United Kingdom; ^2^ MRC Centre for Drug Safety Science, Department of Clinical and Molecular Pharmacology, Institute of Translational Medicine, University of Liverpool, Liverpool, United Kingdom

**Keywords:** NRF2, miRNA, transcription factor, oncogenesis, drug resistance

## Abstract

Nuclear factor (erythroid-derived 2)-like 2 (NRF2) is a key transcription factor that regulates the expression of over a hundred cytoprotective and antioxidant genes that provide cellular protection from reactive oxygen species. Chemotherapy resistance in several cancers has been linked to dysregulation of the NRF2 signalling pathway, moreover there is growing evidence that NRF2 may contribute to tumorigenesis. MicroRNA (miRNA) are small non-coding RNA sequences that post-transcriptionally regulate mRNA sequences. In cancer pathogenesis, aberrantly expressed miRNAs can act as either tumor suppressor or oncogenic miRNA. Recent evidence has been described that identifies a number of miRNA that can be regulated by NRF2. This review outlines the importance of NRF2 in regulating miRNA, and the functional role this may have in the tumorigenesis of human malignancies and their chemotherapy resistance.

## INTRODUCTION

Nuclear factor (erythroid-derived 2)-like 2 (NRF2 and also known as NFE2L2) is a member of the Cap ‘n’ Collar basic leucine zipper transcription factor family [1, 2]. It plays a major role in protecting the cell from reactive oxygen species (ROS) which can present through many insults including UV light, heavy metals, bacterial infection and pharmacological interventions. NRF2 directly controls the expression of a number of genes involved in regulating cellular antioxidant levels and detoxification, and these genes include haem oxygenase-1 (HO-1), glutathione rate-limiting enzymes (GCLM and GCLC) and, NAD(P)H dehydrogenase quinone (NQO)1. Under basal conditions NRF2 is regulated by its inhibitor Kelch-like ECH-associated protein (KEAP1), whose binding results in NRF2 degradation via a Cul 3 ubiquitination-dependent mechanism [3]. Upon activation via an increase in cellular ROS levels KEAP1 dissociates from NRF2 which then translocates to the nucleus under the control of its nuclear localisation signal and binds to the antioxidant response element (ARE). This in combination with small MAF co-factors induces the transcription of NRF2-regulated genes [4].

Despite the long established cytoprotective role of NRF2, there is growing evidence to suggest the NRF2 response can be hijacked by cancerous cells to aid chemotherapy resistance. There is an increasing recognition that NRF2 may protect cancerous cells via the over-production of antioxidants and detoxification proteins [5-7]. Furthermore NRF2 has been reported to up-regulate the expression of drug efflux pumps, such as ABCG2 in lung cancer, which play a crucial role in protecting the cancerous cells from clinically used chemotherapy agents. [8]. Cancers affected by the chemo-protective NRF2 activity include solid tumors, such as; breast, lung, and liver; as well as haematological malignancies, such as; chronic lymphocytic leukaemia (CLL) and acute myeloid leukaemia (AML) [9-11]. High basal NRF2 levels are thought, to be predominantly due to mutations in either NRF2 itself, or its inhibitor KEAP1; preventing NRF2/KEAP1 binding and thereby protecting NRF2 against ubiquitination and degradation [9]. More recently three groups have shown that high NRF2 activity occurs as a consequence of deregulation of the transcription of NRF2 and not somatic mutations in either NRF2 and or its inhibitor KEAP1 [12-14].

**Figure 1:**
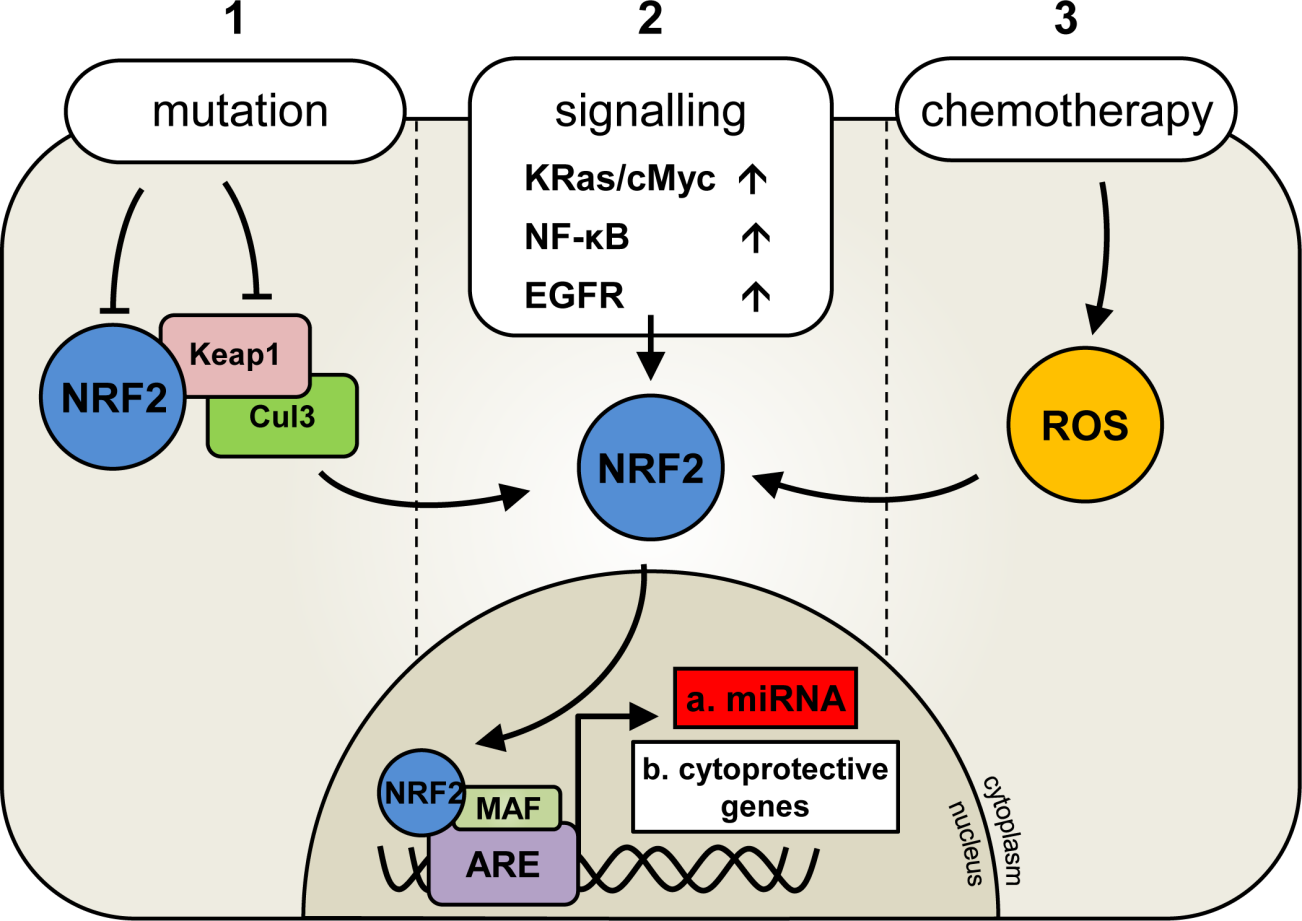
Deregulation of Nrf2 activation in human malignancies Under normal conditions NRF2 transcription factor activity is regulated by its inhibitor KEAP1which binds to NRF2 and targets it for degradation via Cul3 ligase and ubiquitination. In the presence of oxidative stress KEAP1 disassociates from Nrf2 allowing its nuclear translocation and subsequent activation. In human cancer, three reported mechanisms of NRF2 activation are important for cancer cell survival and are outline (1, 2 or 3).

In addition to its involvement in chemo-resistance, there is evidence to suggest that NRF2 may actively promote tumorigenesis and cell survival when activated in these cancers. For example; NRF2 has recently been shown to induce the expression of the anti-apoptotic BCL2 gene which is associated with poor prognosis in AML and colon cancer [15-17]. Furthermore NRF2 has been reported to be activated by the oncogenes KRAS and BRAF in both lung and pancreatic cancer cells [12]. These studies provide evidence for a unique and dynamic role for NRF2 in the development and protection of human cancer cells through a finely balanced system of intracellular effects (Figure 1).

In light of the rapid pace of research into cancer-associated oncogenes, tumor suppressor genes and chemo-preventative genes, and the growing interest in NRF2 in these processes, this review explores the relationship between NRF2 and its transcription factor control of a group of non-coding RNA molecules that regulate gene expression post-transcriptionally. These small non-coding RNAs are known as microRNA (miRNA) and are thought to fine tune normal cellular processes by regulating the expression of mRNA. In cancer pathogenesis, aberrantly expressed miRNAs can act as either tumor suppressor miRNA or oncogenes ‘oncomiRs’.

MicroRNA are under the transcriptional control of transcription factors that predominantly bind to sites within 1 kb upstream of the pre-miRNA start site. NF-κB for example has shown to regulate miR-34a by binding to a site -149 bp from the miRNA start site [18, 19]. It is only recently that microRNA have been shown to be regulated by NRF2. ChIP sequencing (ChIP-seq) data has provided evidence that NRF2 can bind to the promoter of several miRNAs and regulate their transcription [20]. Furthermore, Singh et al identified two miRNA (miR-1 and miR-206) that are indirectly regulated by NRF2 [21]. The potential of NRF2-regulated miRNA, and their ability to target cancer-associated genes, is presently largely undefined.

### Transcriptional regulation of miRNA

miRNA are first transcribed in the nucleus by RNA polymerase II or III as pri-miRNA, from here the enzyme Drosha cleaves the pri-miRNA becoming pre-miRNA. The pre-miRNA undergo cytoplasmic translocation in a process mediated by Exportin-5. Here the pre-miRNA is cleaved by the Dicer/TRBP complex resulting in the formation of a single mature strand of miRNA. The mature miRNA form part of an RNA-induced silencing complex (RISC), which allows the miRNA to bind to the 3' UTR of their target mRNA through complimentary base pairing. A high degree of complementarity results in the degradation of the target mRNA, while lower levels of complementarity will block mRNA translation. In mammals it is the latter process that generally predominates [22].

### Tumor Suppressor miRNA and oncomiRs

**Figure 2:**
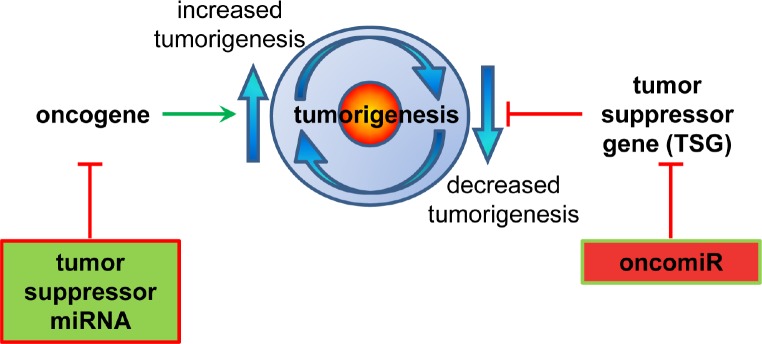
miRNA regulation of the processes involved in tumorigenesis Schematic diagram depicting the ways in which oncogenes can increase, and tumor suppressor genes (TSG) can decrease the potential of tumor generation. miRNAs suppress gene function. A tumor suppressor miRNA would suppress an oncogene's function, and conversely an oncomiR miRNA would suppress a TSG gene's function.

miR targeting of oncogene mRNA expression can act in its nature as a tumor suppressor (Figure 2); for example, miR-15a, which is downregulated in CLL, prostate cancer, and pituitary adenomas, targets anti-apoptotic BCL2 [23, 24]. Conversely, an ‘oncomiR’ is an miRNA that targets directly tumor suppressor mRNA expression. miR-21, for example, is up-regulated in a range of malignancies; including solid tumors, CLL, AML, and multiple myeloma (MM); through targeting the classic tumor suppressor PTEN [23, 25].

The functional role of these miRNAs in malignancies appears to be frequently tumor specific. For example the lin4 ortholog miR-125b acts as an oncomiR in several AML subtypes. It is upregulated 90-fold in the leukaemic translocation t(2;11)(p21;q23), and when ectopically over-expressed in mice causes leukaemia (the specific subtype of AML depends on the level of miR-125b expressed) [26, 27]. In breast cancer however, miR-125b acts as a tumor suppressor by down-regulating oncogenes such as ETS1 (which is required for angiogenesis and metastasis) [28].

### NRF2 regulated miRNA and there relevance to human cancer

NRF2 plays a dual role in human malignancies. Its cytoprotective function protects cells from oxidative damage thereby acting as a tumor suppressor; its deregulation however can protect cancerous cells from ROS damage (primarily induced by treatment with chemotherapy agents), thus acting as an oncogene [9]. Its apparently central and important roles in cancer development and resistance makes it an attractive therapeutic target.

Although the NRF2 signalling pathway has been well studied, less is known about the role of miRNAs in the regulation of NRF2 expression. Bioinformatics databases, such as miRbase (http://www.mirbase.org/) or miRNA.org http://www.microrna.org, identify a number of miRNAs predicted to target the NRF2 transcript. Of these, only miR-28 and miR-144a have been experimentally proven to directly target and repress NRF2 mRNA [29, 30]. Interestingly, NRF2 can also be indirectly regulated by miRNA; through the targeting and repression of its endogenous inhibitor, KEAP1, via miR-200a. This serves to highlight the potential level of complexity afforded through the exploration of miRNA-driven NRF2-regulation.

### NRF2 as a miRNA transcription factor

Despite its function as a transcription factor for classic cytoprotective genes, little literature can be found exploring the possibility of miRNA transcript regulation by NRF2. One key study performed chromatin immunoprecipitation sequencing (ChIP-Seq) on lymphoid cells treated with the NRF2 activator, sulforaphane, in order to identify NRF2 target transcripts, including miRNAs [20]. High confidence ChIP-Seq peaks were identified in the vicinity of nine putative miRNAs, indicating they are regulated by NRF2 (Table 1). Conformational studies revealed, however, that only miR-365, miR-193b, miR-181c, and miR-29b were expressed in the lymphoblast cell line; and only miR-29b showed a significant change in expression in response to NRF2 activation. Another recent study by Singh et al shows that NRF2 can indirectly downregulate miR-1 and miR-206 through its direct regulation of HDAC4 expression [21]. They showed that both miRNA regulate various glucose metabolism genes such as transketolase (TKT) and glucose-6-phosphate dehydrogenase (G6PD). In cancer miR-1 and miR-206 are repressed in various cancers as their primary targets are considered tumor promoting, hence miR-1 and miR-206 are considered tumor suppressor miRNAs [21]. To date, this is the only data available to suggest that NRF2 is responsible for miRNA transcription. Using these studies, we will explore the relevance of these miRNAs in the pathogenesis of malignancies, as driven by NRF2 transcription factor activity.

**Table 1:** NRF2-regulated miRNA Chromosome location and established gene targets and whether these targets can act as an oncogenic or tumour suppressor gene (TSG) miRNA.

miRNA	Location	Targets	References
miR 193b/365	Chr16, 14397824-14397906 (miR193b) 14403142-14403228 (miR-365)	TTf1 - oncogenic BCL2 – TSG Cyclin D- TSG, uPa	(Qi et al., 2012) (Nie et al., 2012) (Li et al., 2009)
miR-29b	Chr7, 130562218-130562298	Sp-1 MCL-1 - oncogenic TCL1 – oncogenic	(Amodio et al., 2012) (Mott et al., 2007) (Pekarsky et al., 2006)
miR-181c	Chr19, 13985513-13985622	SIRT1- oncogenic and TSG KRAS - oncogenic TGFβ - TSG TNF - TSG NOTCH - oncogenic and TSG	(Zhang et al., 2012) (Schonrock et al., 2012) (Hashimoto et al., 2010)
miR-617	Chr12, 81226312-81226408	N/A	
miR-592	Chr7, 126698142-126698238	N/A	
miR-1207	Chr8 129061398-129061484.	HBEGF	(Papagregoriou et al., 2012)
miR-32	Chr9- 111808509-111808578	PIK3IP1 - TSG BTG2 – TSG	(Jalava et al., 2012)
miR-200c	Chr12, 7072862-7072929	ZEB1, FHOD1, PPM1F, TUBB3-TBK1, BMI1-oncogenic PPP2R1B - TSG	(Burk et al., 2008) (Jurmeister et al., 2012) (Cochrane et al., 2010) (Lin et al., 2012)
miR-550	Chr7, 30329410-30329506	CPEB4	(Tian et al., 2012)

### miR-193b-365 cluster

Aberrant expression of the miR-365/193b cluster has been linked to malignancies such as MM, lung cancer, and colon cancer. For example, the cluster was found to be up-regulated in a range of MM cell lines and primary samples [31]. Interestingly, both miR-365 and miR-193b can be tumor associated without the other member of the cluster. In lung cancer, down-regulation of miR-365 has been implicated in increased expression of thyroid transcription factor 1 (TTf1), important for lung development and commonly up-regulated in lung tumors [32]. Conversely, in colon cancer, up-regulation of miR-365 is thought to repress tumor formation and maintenance by targeting the anti-apoptotic genes BCL-2 and cyclin D1. Downregulated miR-365 in colon cancer increases sensitivity to 5-fluorouracil [33]. miR-193b is also down-regulated in breast cancer cell lines, where it is thought to repress oncogenic expression of urokinase-type plasminogen activator (uPA) [34-36]. Finally ChIP-Seq data indicates that NRF2 can regulate the miR-365/193b cluster [20]. However the differing roles of the miRNA described in this cluster suggest a complicated system of regulation that is only befiniing to be understood in human cancer.

To date, in both multiple myeloma and colon cancer insufficient research has been undertaken to characterise the role played by NRF2 in these cancers. Nevertheless NRF2 plays a unique role in chemotherapy resistance through which is highlighted in many experiments and several types of malignancy. This is because NRF2 is up-regulated in response to front-line chemotherapy agents including cyatarbine/daunorubicin, bortezomib (myeloma) and 5-fluorouracil (colon cancer), resulting in the induction of cytoprotective genes, thereby contributing to the decrease in the cell's sensitivity to these drugs [37-39]. However whether the NRF2 regulated cytoprotective genes already characterised are responsible for this chemotherapy resistance or its due to in part to miRNA regulated by NRF2 is unclear. If NRF2 is indeed responsible for the clusters downregulation in colon and lung cancer, it would concur with NRF2's role being in protection against cell damage and promoting cell survival (i.e. by preventing BCL-2 inhibition) and by allowing TTf1 upregulation. Interestingly the cluster is upregulated in MM, where the functional role of this miRNA is yet to be determined.

### miR-29b

The miR-29 family are commonly regarded as tumor suppressor miRs and are down-regulated in the majority of cancers with which they are associated (prostate and MM)[40]. For example, one study in MM identified a negative feedback loop between miR-29b and a putative target, transcription factor SP-1. Up-regulation of SP-1 is observed in MM, thus, artificial overexpression of miR-29b results in a reduction of SP-1-driven growth and survival of MM cells, which is further enhanced in combination with front-line MM chemotherapy bortezomib [40]. Furthermore SP-1 binding sites have been located on the murine NRF2 promoter, possibly indicating the existence of a feedback loop; NRF2 down-regulating miR-29b results in increased SP-1 upregulation which in turn up-regulates NRF2 [41]. NRF2 repressing miR-29b, could therefore allow SP-1 and NRF2 up-regulation thereby protecting against apoptosis. The function of miR-29b is important in human cancer and is in much need of further experimentation and to make clear the role of transcription factors like NRF2, SP1 and NF-κB in its regulation.

### miR-181c

miR-181c is a member of the miR-181 family which is predominantly associated with hematopoietic cell differentiation and cancer pathogenesis. miR-181c is highly expressed in the thymus, brain, liver and the bone marrow [42], and has been shown to repress a range of cancer-associated genes including tumor suppressor TGF-β1, oncogenic histone deacetylase SIRT1, pro-inflammatory TNF, highly conserved developmental regulator NOTCH, and oncogenic GTPase KRAS [43-45]. The function of miR-181c in cancer appears to be tumor type-specific. For example; down-regulation of miR-181c was observed in the majority of gastric cancer samples tested, however a small proportion showed a significant increase in expression indicating an ability to act as both an oncomiR and tumor suppressor miR [45]. As miR-181c was shown to repress oncogenes NOTCH and KRAS it is likely that its primary function in gastric cancer is as a tumor suppressor miR, hence it's more frequent down-regulation. Down-regulation of miR-181c seems consistent with studies carried out in glioblastoma primary tissue and cell lines [46], however work on liver carcinoma has identified cell-type dependant expression patterns of miR-181c [47]. Interestingly, miR-181c is up-regulated in hepatic stem cell-like carcinoma, but down-regulated in mature hepatocyte carcinoma, indicating that it may play an important role maintaining the stem cell like nature of cancerous cells.

Loss of miR-181c, and other member of the miR-181 family, appears to contribute to several cancer profiles, perhaps due to its role in cell differentiation along various lineages [42, 48, 49]. While NRF2 deficiency can cause gastric cancer in xenograft models, little work has been done on its role in gastric cancer itself. Upregulation of the NRF2 target HO-1 however has been reported to increase resistance to cisplatin treatment [50]. NRF2 can again regulate glioblastoma apoptosis via HO-1 expression [51]. Studies in hepatocyte carcinoma linked p62 accumulation (a marker for autophagy dysregulation) with NRF2 upregulation. P62 can bind to the NRF2 binding sites on the NRF2 inhibitor Keap1, resulting in NRF2 stabilisation and its binding to the ARE [52]. The ChIP-Seq data raises the possibility that NRF2 controls miR-181c expression, if so the down-regulation of this miRNA in the aforementioned disorders would promote the activation of various pro-survival pathways.

### miR-32

miR-32 is considered an oncomiR, and, as such, is often up-regulated in associated malignancies. In castration-resistant prostate cancer, for example, miR-32 is up-regulated by androgen, targets PI3K inhibitor PIK3IP1, and tumor suppressor BTG2 [53]. It's up-regulation in prostate cancer is associated with increased severity, possibly due to the downregulation in BTG2. BTG2 is an anti-proliferative gene and is downregulated in a variety of cancers, its decreased expression increases the proliferative and survival nature of these cells [53].

miR-32 up-regulation was also found in both malignant mesothelioma, and renal cell carcinoma [54, 55]. As in prostate cancer, in renal cell carcinoma high expression of miR-32 was linked to poorer prognosis, therefore suggesting a potential role as a prognostic biomarker. However, no putative targets were suggested for miR-32 in this cancer subtype [55].

In prostate cancer NRF2 upregulation is primarily due to point mutations in Keap1, and results in resistance to commonly used chemotherapy paclitaxel. Furthermore NRF2 shRNA was shown to reduce tumor size *in vitro* and *in vivo*, indicating NRF2 plays a role in proliferation in prostate cancer [56]. Conversely BTG2 has been reported to associate with NRF2 (as a co-activator) and up-regulate antioxidant genes in a NRF2-dependent manner [57].

Like prostate cancer NRF2 is also up-regulated in some renal cell carcinoma subtypes. In heredity type 2 papillary renal cell carcinoma, the mechanism by which NRF2 is activated differs depending on whether the disease is heredity or sporadic. Patients with the heredity disorder generally have a causative mutation in fumarate hydratase (FH), which results in intracellular fumarate accumulation. Fumarate alters Keap1, preventing NRF2/Keap1 interactions, thereby constitutively activating NRF2 [58]. Sporadic mutation patients also have dysregulated NRF2, but generally lack an FH mutation. In these cases constitutively activated NRF2 is due to mutations in NRF2, Cul3 or Sirt1. Furthermore NRF2 activation was believed to promote tumorigenesis in this cancer [59].

NRF2 regulation of miR-32 may be responsible for miR-32s upregulation in the aforementioned malignancies. For example in prostate cancer miR-32 represses PI3K inhibitors, this would allow activation of PI3K which has been reported to up-regulate NRF2. The NRF2 dependent induction of miR-32 could therefore be another mechanism by which NRF2 can become up-regulated via this positive feedback loop [60]. Overall this would support our current knowledge of NRF2s ability to promote cellular survival.

### miR-200c

miR-200c is considered a tumor suppressor miR due to its ability to inhibit epithelial to mesenchymal transition (EMT), and is involved in a number of cancers such as breast, ovarian, endometrial, colorectal cancer, and pancreatic cancer [61-63]. miR-200c can directly regulate EMT. EMT is the process by which cells are able to detach from one another and become mobile, the cells also change phenotype and are able to produce pro-invasive molecules such as proteases, allowing them to break down and pass through the basement membrane. The process of EMT is used by tumors to metastasise and invade other tissues and organs. One important activator of EMT is ZEB1 (which repress genes such as E-cadherin, allowing EMT to occur). Mouse studies indicate ZEB1 promotes cancer metastasis [64, 65]. ZEB1 inhibits miR-200c expression, while miR-200c in turn inhibits EMT by targeting ZEB1 thereby creating a negative feedback loop [66]. In breast cancer, miR-200c has also been reported to repress EMT independently of ZEB1, by silencing FHOD1, and PPM1F (both play a key role in remodelling the actin cytoskeleton from an epithelial to a mesenchymal phenotype) [67]. This further suggests that enhancing miR-200c expression could be a viable option to limit metastases.

miR-200c has also been linked to drug resistance in several malignancies. For instance, breast and ovarian cancer cells with higher levels of miR-200c expression are more sensitive to the chemotherapy agent paclitaxel, due to targeting of microtubule component, TUBB3 [61]. Similarly, studies have shown that breast cancer cells with low miR-200c expression demonstrate increased resistance to radiotherapy, whilst, when over-expressed, this miRNA is responsible for increased apoptosis [68]. This increased sensitivity is partly due to miR-200c targeting TBK1, which in cancers represses apoptosis and activates other oncogenic pathways such as AKT. It should be noted that other targets of miR-200c (i.e. TUBB3 and BMI1) are also likely to be involved in this process.

Chemo-resistance due to the down-regulation of miR-200c has also been seen in melanoma, due to its reduced targeting of oncogenic cell cycle regulator BMI1; and small cell lung cancer, in which increased miR-200c is able to improve sensitivity to cisplatin and cetuximab chemotherapies [69, 70]. Conversely miR-200c overexpression has been reported to cause chemo-resistance and is linked with poor prognosis in esophageal cancers, due to increased AKT activation via miR-200c targeting the AKT phosphorylation repressor PPP2R1B [71]. These conflicting findings suggest a tissue-type specific role for miR-200c in cancer.

Since ZEB1 inhibits E-cadherin allowing EMT to occur and that E-cadherin has been shown to prevent the accumulation of NRF2 in the nucleus and that inhibition of E-cadherin results in the upregulation of NRF2 [72], the downregulation of miR-200c by NRF2 could potentially provide a mechanism by which NRF2 indirectly regulates E-cadherin and therefore metastasis.

In ovarian cancer NRF2 upregulation is again linked to drug resistance against the platinum compounds used to treat this disorder. This upregulation was primarily due to mutations in Keap1 [73]. Unlike ovarian cancers, NRF2 is downregulated in a significant proportion of breast cancer patient samples, due to Cul3 overexpression [74]. Conversely NRF2 is up-regulated in tamoxifan resistant breast cancer cells, and disruption of NRF2 increases sensitivity to tamoxifan [75]. This indicates the role of NRF2 signalling in breast cancer is complex. As previously stated miR-200c downregulation in both breast and ovarian cancers indirectly results in AKT upregulation. The PI3K/AKT pathway are known NRF2 activators, therefore NRF2 regulating miR-200c could potentially provide a feedback mechanism [60]. miR-200c is downregulated in both breast and ovarian cancer, yet NRF2 is only upregulated in ovarian cancer again suggesting the interaction is tissue specific and that other transcription factors are likely to be involved.

### miR-1 and miR-206

Mir-1 and its paralog miR-206 are tumor suppressor miRs which are indirectly regulated by NRF2 via HDAC4. miR-1 and miR-206 repress glucose metabolism genes such as TKDT and G6PD. In cancers characterised by high levels of NRF2, HDAC4 localises in the nucleus and represses the expression of these miRNA, resulting in increased nucleic acid and lipid synthesis, thereby providing a mechanism by which NRF2 can increase cellular proliferation in these cancer cells. This is supported by *in vivo* studies where overexpression of either miR-1 or miR-206 reduced lung cancer growth in nude mice [76]. As expected both miRNA are downregulated in a range of cancers, for example miR-1 is downregulated in hepatocellular carcinoma and colorectal cancer [77, 78], while miR-206 is downregulated in gastric and breast cancer [79, 80]. Taken together, this data suggests a dynamic role for NRF2 in regulating these two miRNA in human cancer.

Interestingly, the miR-1 target endothelin-1 (ET-1) is a growth promoting peptide that plays an oncogenic role in hepatocellular carcinoma by significantly increasing its ability to proliferate [77]. miR-1 also targets the tyrosine kinase receptor c-MET, c-MET encodes a hepatocyte growth factor (HGF) receptor which when bound activates various oncogenic pathways including PI3K, RAS and CDC42 thereby promoting survival, proliferation and mobility [81]. The c-MET signalling pathway is upregulated in both hepatocellular carcinoma and colorectal cancer [78, 81]. *In vivo* studies in hepatocellular carcinoma has shown c-MET constitutive overexpression is required to drive these tumors, and inhibiting c-MET expression negatively affected the tumor [81, 82]. Furthermore patients characterised with high levels of MET are associated with poorer prognosis and increased likelihood of cancer metastasis [83]. Since NRF2 is found to be upregulated in both colorectal and hepatocellular carcinoma and miR-1 is inhibited then this forms the hypothesis that NRF2 could be linked to high c-MET expression in these diseases.

The downregulation of miR-206 in breast cancer is correlated with larger tumor size, but when miR-206 is ectopically overexpressed colony formation and cellular proliferation is inhibited by the prevention of G1 to S cell cycle transition (via the miR-206 target Cyclin D2) [80]. Similarly gastric cancer is characterised by elevated cyclin D2 levels and low miR-206 expression. The increased proliferation from cyclin D overexpression is inhibited when miR-206 is upregulated [84]. Therefore, NRF2 could be indirectly involved in regulating cell proliferation through its association with HDAC4 and miR-206 and ultimately cyclin D1 overexpression.

### miRNAs- miR-617, -592, -1207 and -550

The miRNAs discussed above have been characterised and many of their targets validated in cancer. The NRF2 regulated miRNAs discussed in this section have emerging profiles in cancer biology.

The role miR-617 plays in cancer is more elusive than many miRNAs, two studies have shown varying results. One of which shows that it is downregulated in adenocarcinoma [85] and the other study into esophageal cancer found miR-617 expression post-cisplatin and 5-fluorouricil treatment to be up-regulated [86]. No experimentally verified targets of miR-617 have currently been identified As NRF2 is activated in response to cisplatin treatment and to 5-fluorouracil treatment [87], this could possibly indicate NRF2 up-regulates miR-617 to protect the cells from damage and apoptosis.

miR-592 is located on chromosome 7 at position 126698142-126698238 and has been associated with a number of malignancies including, colorectal and liver carcinoma. [88, 89]. For example, in colorectal cancer, low levels of miR-592 expression have shown promise as a biomarker, identifying patients who will react poorly to anti-EGFR chemotherapies [88]. Interestingly, miR-592 down-regulation has also been observed in colon cancers with deficient DNA mismatch repair (dMMR), a common mutation in colorectal tumors. [90]. miR-592 was also down-regulated in hepatitis B hepatocellular carcinoma [89]. NRF2 mediated drug resistance has been shown in colon cancer and is up-regulated in hepatocyte carcinoma [38, 52], indicating a possible link between the downregulation of miR-592 in these disorders and NRF2 activity.

While miR-1207 may be transcribed by NRF2, there have been few studies on its role in malignancies, therefore making its role in cancers elusive. It is located on chromosome 8 129061398-129061484. It has been reported to be up-regulated in prostate cancer, with levels varying depending on disease risk [91]. It has also been shown to be highly expressed in colon cancer. [92, 93]. Since NRF2 is upregulated in prostate cancer, raising the possibility of high levels NRF2 being responsible for the upregulation of this miRNA in both prostate and colon cancer.

Like miR-1207, and miR-617, little work has been done to specifically identify the role of miR-550 in malignancies. miR-550 is located in chromosome 7 at 30329410-30329506 and was found to be overexpressed in MALT lymphoma [94], and in pituitary gland tumors post-treatment with bromocriptine, a front-line dopamine agonist [95]. miR-550 predicted targets (according to DIANA-microT V3.0) included the tumor suppressor BCL11B again suggesting NRF2 up-regulation of this miRNA to promote cellular survival.

### 3' and 5' sequence analysis of miRNA regulated by NRF2

To further detail the role of NRF2 in regulating the miRNA described in this review paper we have examined the sequences directly upstream and downstream, of each miRNA described, for ARE binding sites. Two web-based transcription factor search programs were used: (1) P-match and (2) TFSEARCH:

1. www.gene-regulation.com/cgi-bin/pub/programs/pmatch/bin/p-match.cgi
2. www.cbrc.jp/research/db/TFSEARCH.html

**Figure 3:**
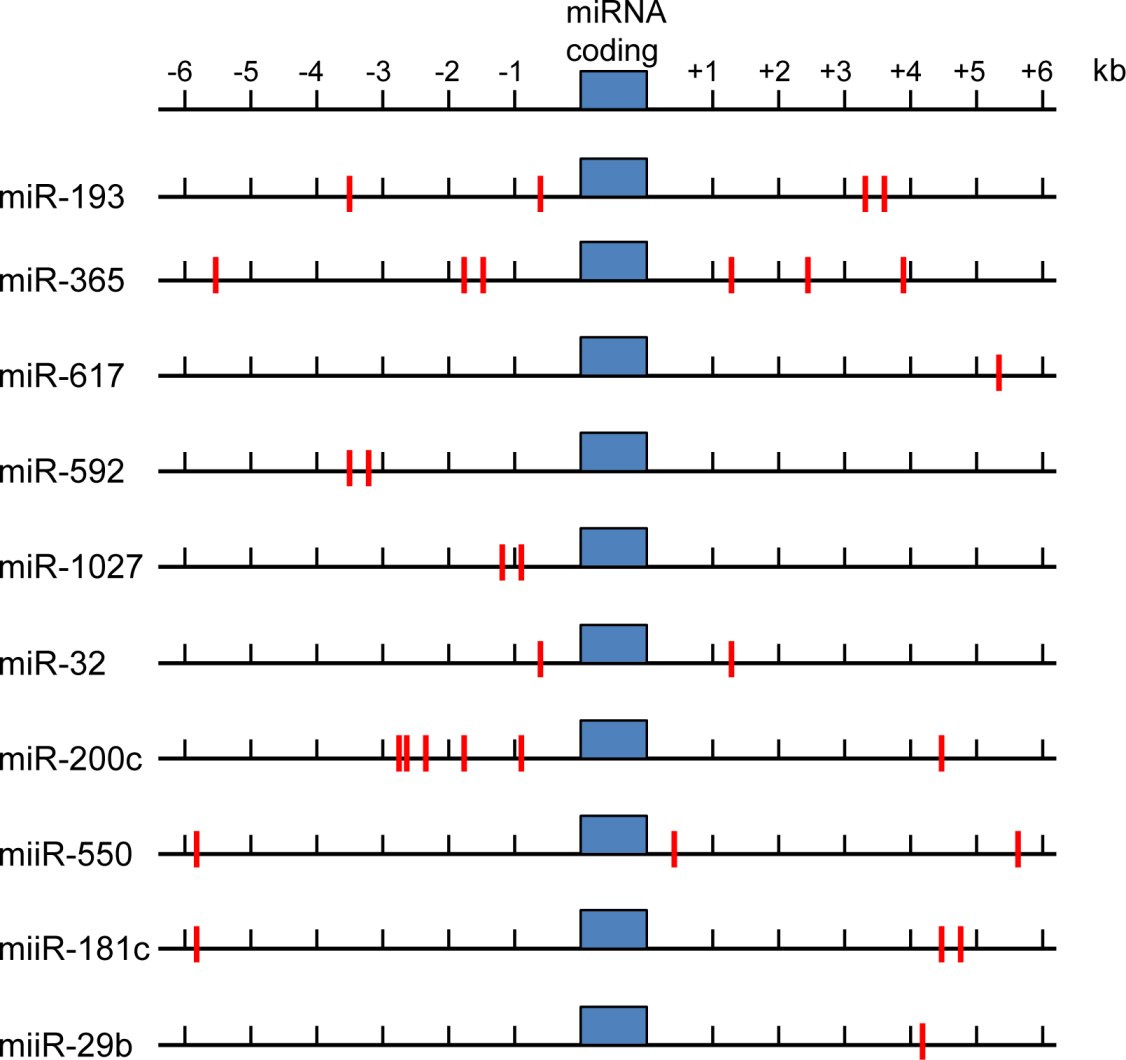
ARE sequences in upstream and downstream regions of NRF2 target miRNA sequences Indicated miRNA coding sequences showing predicted ARE sites (red lines) as NRF2 binding sites.

ARE matches were cross-checked between programs and presented in Figure 3. It is important to note that the main purpose of this bioinformatic approach was to determine ARE binding sites within the 3' and 5' sequence of candidate NRF2 regulated miRNA.

Figure 3 highlights the number of ARE sites located within the proximity of the highlighted miRNA. Whether this constitutes the promoter region of these miRNA is open to speculation. However further examination and verification of the proximal miRNA regions for RNA polymerase 2 (pol2) binding sites using ChIP-seq data from the ENCODE consortium was performed (ENCODE - data were generated by the laboratory of Ruan using the human promyelocytic cell line NB4 http://genome.ucsc.edu/ENCODE/). This was then cross-referenced to histone H3K27ac binding (which are often found in active regulatory elements) [96, 97]. Our preliminary searches found a high degree of pol2 and H3K27ac activity in the proximal regions of the miRNA listed in Figure 3. Taken together, this suggest a promoter element within the miRNA proximal sequences and therefore further implicates NRF2 as the transcription factor capable of regulating the expression of these miRNA.

## CONCLUSION

This review has discussed the potential for NRF2 to regulate miRNA expression. Moreover, we reasoned the regulatory role that NRF2 could have on these miRNAs in human cancer. Several miRNAs discussed seem to have conflicting roles in different cancer types, perhaps suggesting that their regulation may be tissue specific and ultimately dependent on the wider context of such tumor specific biology. This implies the involvement of other transcription factors in their regulatory network. We analysed these 3' and 5' sequences located close to the miRNA and found sites specific for NRF2 binding, however more functional studies need to be undertaken to confirm NRF2 ability to regulate these miRNAs. This will help define a relationship between NRF2 levels and miRNA expression in human cancer and inform drug development and therapeutic strategies moving forward._*e*_
